# Coexpression analysis of nine neuropeptides in the neurosecretory preoptic area of larval zebrafish

**DOI:** 10.3389/fnana.2015.00002

**Published:** 2015-02-12

**Authors:** Ulrich Herget, Soojin Ryu

**Affiliations:** ^1^Developmental Genetics of the Nervous System, Max Planck Institute for Medical ResearchHeidelberg, Germany; ^2^The Hartmut Hoffmann-Berling International Graduate School of Molecular and Cellular Biology, University of HeidelbergHeidelberg, Germany

**Keywords:** neuroendocrine system, hypothalamus, preoptic region, paraventricular nucleus, zebrafish, coexpression

## Abstract

The paraventricular nucleus (PVN) of the hypothalamus in mammals coordinates neuroendocrine, autonomic and behavioral responses pivotal for homeostasis and the stress response. A large amount of studies in rodents has documented that the PVN contains diverse neuronal cell types which can be identified by the expression of distinct secretory neuropeptides. Interestingly, PVN cell types often coexpress multiple neuropeptides whose relative coexpression levels are subject to environment-induced plasticity. Due to their small size and transparency, zebrafish larvae offer the possibility to comprehensively study the development and plasticity of the PVN in large groups of intact animals, yet important anatomical information about the larval zebrafish PVN-homologous region has been missing. Therefore we recently defined the location and borders of the larval neurosecretory preoptic area (NPO) as the PVN-homologous region in larval zebrafish based on transcription factor expression and cell type clustering. To identify distinct cell types present in the larval NPO, we also generated a comprehensive 3D map of 9 zebrafish homologs of typical neuropeptides found in the mammalian PVN (arginine vasopressin (AVP), corticotropin-releasing hormone (CRH), proenkephalin a (penka)/b (penkb), neurotensin (NTS), oxytocin (OXT), vasoactive intestinal peptide (VIP), cholecystokinin (CCK), and somatostatin (SST)). Here we extend this chemoarchitectural map to include the degrees of coexpression of two neuropeptides in the same cell by performing systematic pairwise comparisons. Our results allowed the subclassification of NPO cell types, and differences in variability of coexpression profiles suggest potential targets of biochemical plasticity. Thus, this work provides an important basis for the analysis of the development, function, and plasticity of the primary neuroendocrine brain region in larval zebrafish.

## Introduction

The vertebrate neuroendocrine system is controlled by specific hypothalamic nuclei. The most extensively studied ones are the paraventricular nucleus (PVN) and supraoptic nucleus (SON) in rodents, which contain neuroendocrine cells expressing secretory neuropeptides. These peptides are transported to the pituitary gland, where they are directly released into the general circulation via the neurohypophysis, or released into the portal system of the adenohypophysis, where they trigger the release of hormones into the general circulation. The two peptides released in the neurohypophsis are arginine vasopressin (AVP), and oxytocin (OXT), and they are produced by distinct magnocellular neurons. Releasing and inhibiting factors acting on adenohypophyseal secretion are instead produced in parvocellular neurons, and include corticotropin-releasing hormone (CRH), thyrotropin-releasing hormone (TRH), methionine or leucine enkephalin (mENK/lENK), neurotensin (NTS), vasoactive intestinal peptide (VIP), cholecystokinin (CCK), and somatostatin (SST) (Swanson and Sawchenko, 1983; Mezey et al., 1985; Simmons and Swanson, 2009).

Coexpression of several of these peptides in the same cell is a common phenomenon in the mammalian PVN. For example, AVP colocalizes with mENK in neurohypophysial terminals in the rat (Martin and Voigt, 1981). Also in rats, CRH can be coexpressed with AVP, OXT, NTS, ENK, VIP, and/or CCK (Burlet et al., 1983; Hökfelt et al., 1983; Roth et al., 1983; Sawchenko et al., 1984a; Mezey et al., 1985; Piekut and Joseph, 1986; Swanson et al., 1986; Sawchenko, 1987; Whitnall and Gainer, 1988; Ceccatelli et al., 1989; Swanson and Simmons, 1989; Arima et al., 2001; Dabrowska et al., 2011). Cells producing OXT were found to also synthesize CRH, CCK, and ENK (Martin and Voigt, 1981; Vanderhaeghen et al., 1981; Rossier et al., 1983; Levin and Sawchenko, 1993). Not all peptide combinations occur, however. The populations of cells producing AVP, OXT, or SST are completely separate in the rat (Swanson and Sawchenko, 1983; Swanson et al., 1986).

The molecular development of the mammalian hypothalamus is largely conserved in zebrafish (Machluf et al., 2011). We have recently defined the location and borders of the larval neurosecretory preoptic area (NPO) as the PVN-homologous region based on transcription factor expression and cell type clustering in larval zebrafish. To identify distinct cell types present in the larval NPO, we also generated a comprehensive 3D map of 9 zebrafish homologs of typical neuropeptides found in the mammalian PVN (*avp*, *crh*, *oxt*, *proenkephalin a* (*penka*), *proenkephalin b* (*penkb*), *nts*, *vip*, *cck*, and *somatostatin* (*sst1.1*); Herget et al., 2014). The larval NPO featured 9 out of 10 peptides also found in the mammalian PVN. One exception was *trh*, which was found to be outside the boundaries of the larval NPO as we defined them. We found a small group of cells producing *cck* at the rostral border of the NPO and cells producing *avp*, *oxt*, *crh*, *penka*, *nts*, or *sst1.1* as dense and intermingled clusters. In contrast, cells producing *penkb* or *vip* appeared to reside in separate subregions of the NPO.

Several of these neuropeptides are coexpressed in the same cells in the mammalian PVN, and extensive coexpression also in the larval NPO seemed likely based on the spatial proximity of cells after 3D registration. We reasoned that definitive classification of distinct cell types cannot be assigned in the larval NPO based on the expression of one neuropeptide alone. Therefore, we analyzed in this study the degree of coexpression of two neuropeptides in the same cell by performing systematic pairwise comparisons of coexpression of *avp*, *crh*, *oxt*, *penka*, *penkb*, *nts*, *cck*, *vip*, and *sst1.1* in the larval zebrafish NPO. Our results show that many of the peptides produced by densely intermingled cells of the larval zebrafish NPO are not coexpressed, while some neuropeptide combinations show occasional, low or moderate levels of coexpression. Interestingly we observed high degrees of coexpression for certain neuropeptide combinations such as *avp* + *crh* and *cck* + *penkb*. These results illustrate that information about coexpressed peptides is essential to identify subclasses of cell types and the classification of cell types should not be based on the expression of one peptide alone within the NPO.

Plastic changes in the coexpression profile of a cell allow it to acquire additional regulatory functions. Indeed, in mammals the coexpression properties of PVN cells are subject to stress-induced plasticity, with different types of stress influencing the expression levels of different neuropeptides (Swanson et al., 1986; Harbuz and Lightman, 1989; Swanson, 1991). The larval zebrafish offers an attractive system to dissect the mechanistic basis of such environment-induced plasticity in the hypothalamus because of the possibility to study the architecture, development, and function of distinct neural circuits in intact and transparent animals. The basal characterization of coexpression profiles is a prerequisite to classify and identify distinct NPO cell types and their plasticity in response to environmental challenges.

## Materials and methods

### Zebrafish preparation

Zebrafish maintenance and breeding were carried out under standard conditions at 28.5°C (Westerfield, 2000). To avoid pigmentation, embryos were incubated in 0.2 mM 1-phenyl-2-thiourea (Sigma-Aldrich). AB/TL zebrafish larvae were fixed at 5 days post fertilization (dpf) in 4% paraformaldehyde (PFA, Merck; in phosphate buffered saline (PBS), pH 7.2–7.3) overnight. All animals were raised under constant conditions and fixed quickly to avoid chronic environmental or acute handling stress. On the following day, larvae were washed briefly with PBST (phosphate-buffered saline with 0.1% Triton X-100, Merck and Roth), then dehydrated with increasing methanol (Merck) concentrations (25%, 50%, 75%, 100%, in PBST, 5 min steps), and stored in 100% methanol at −20°C. All procedures were performed according to the guidelines of the German animal welfare law and approved by the local government.

### Whole-mount fluorescent *in situ* hybridization

*In situ* hybridization (ISH) probes for *avp* (Eaton et al., 2008), *oxt* (Unger and Glasgow, 2003), *sst1.1* (Devos et al., 2002), *trh*, *crh* (Löhr et al., 2009), *vip* (Wolf and Ryu, 2013), *cck*, *penka*, *penkb*, and *nts* (Herget et al., 2014) were previously described. Riboprobes were synthesized from linearized plasmids following the instructions provided with the digoxygenin labeling mix (Roche). Fluorescent ISH was performed based on a previously published protocol (Lauter et al., 2011).

### Microscopy and image processing

For imaging, larval heads were cleared in 80% glycerol (Gerbu) in PBS for 1 h. Dorsal confocal stacks of larval heads were recorded using a Leica SP5 confocal microscope with a Nikon 20x glycerol objective. Each channel was recorded sequentially, using alternating excitation wavelengths specific for each tyramide, to reduce interfering signals from overlapping emission spectra. Acquisition settings were adjusted for each stack to obtain the optimal image quality of the desired volume. Stacks were evaluated using Amira 5.4 (Visualization Sciences Group) to create maximum intensity projections that were restricted to the volume of interest, excluding signals from planes above or below. Staining signal was analyzed plane by plane within the NPO. Brightness and contrast were adjusted for each channel. Any accumulation of signal with the proper shape and size of a typical cell was included in the analysis and compared to the signal in co-stained channels at the same location. Thus, coexpression was determined by the spatial overlap of cells stained for different peptide markers. Images of single planes and maximum projections were exported from Amira and arranged into figures using Adobe Illustrator. All images show dorsal views of substacks or single planes, with the rostral direction on the left side, unless indicated otherwise.

## Results

**Figure 1:**
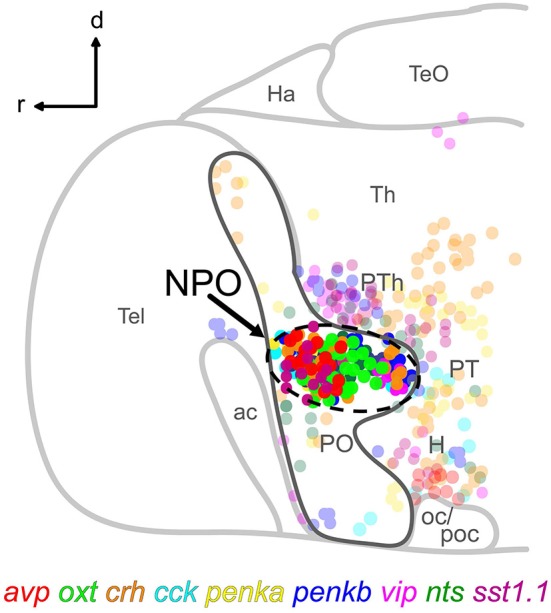
**Schematic lateral view of a 5 dpf larval zebrafish brain showing the location of the NPO (dashed line) within the *otpa*-positive part (dark gray line) of the preoptic area, and the spatial distribution of nine cell types expressing the indicated neuropeptides**. Cells clustering within the NPO are opaque. For more details, the reader is referred to our previously reported chemoarchitectural map (Herget et al., 2014). Abbreviations: ac, anterior commissure; d, dorsal; H, hypothalamus; Ha, habenula; NPO, neurosecretory preoptic area; oc, optic chiasm; PO, preoptic area; poc, postoptic commissure; PT, posterior tuberculum; PTh, prethalamus; r, rostral; Tel, telencephalon; TeO, optic tectum; Th, thalamus.

To comprehensively analyze the degree of coexpression of two peptides in the same cell, we performed cell by cell comparisons of pairwise combinatorial ISH staining of nine peptide markers that we had previously identified to be expressed in the 5 dpf larval NPO. The NPO is defined by the dense clustering of cells expressing these peptides within the transcription factor *orthopedia a (otpa)*-positive preoptic area (Figure 1). Altogether we examined 36 pairwise combinations of neuropeptides, analyzing a minimum of 5 animals per pair, but the sample size varied and was larger for some peptide combinations. All data was obtained from 5 dpf larvae, since the original NPO cell type map was generated for that stage. We found broadly three different categories of coexpression extent: (1) Absence of coexpression in all animals; (2) Occurance of coexpression in a single, few or several cells only in some animals analyzed (“variable coexpression”); (3) Coexpression in several cells in all animals analyzed (“consistent coexpression”).

### No coexpression in all animals

**Figure 2:**
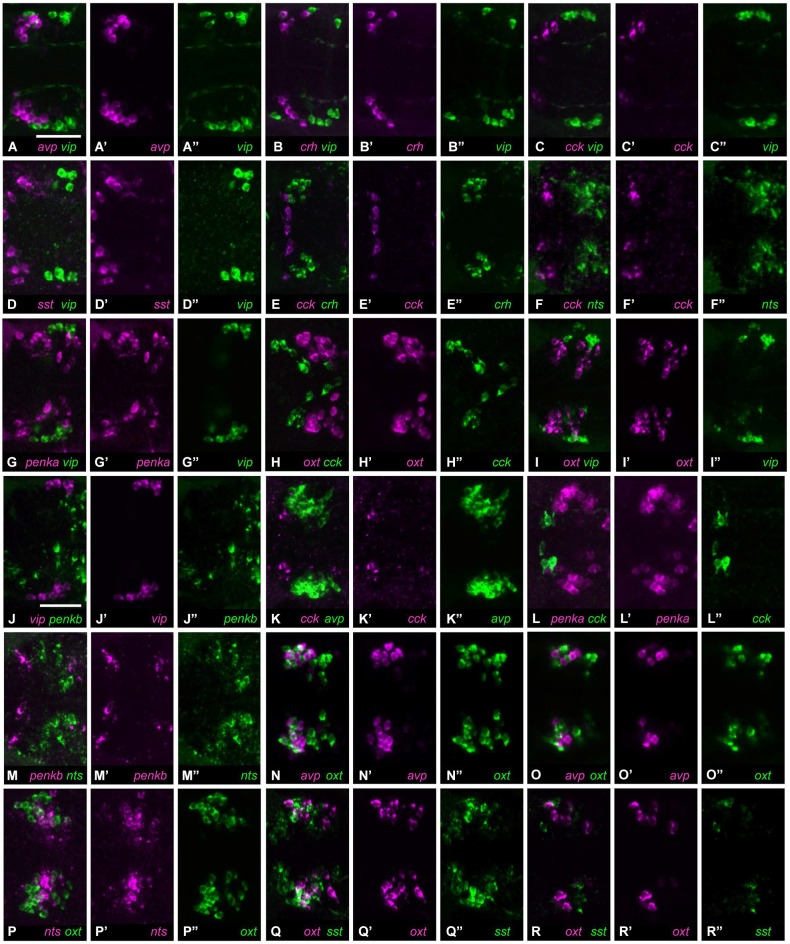
**Cell type staining combinations showing no coexpression. (A)** Cells expressing *avp* **(A’)** or *vip* **(A”)** form neighboring but separate clusters. **(B)** Cells expressing *crh* **(B’)** or *vip* **(B”)** are similarly separated. **(C)** Cells expressing *cck* cluster rostrally **(C’)**, and are therefore distant from *vip*-positive cells **(C”). (D)** Cells expressing *sst1.1* **(D’)** also cluster in a separate region from *vip*-positive cells **(D”). (E)** *cck*-positive cells **(E’)** are rostrally neighboring *crh*-positive cells **(E”). (F)** Cells expressing *cck* **(F’)** or *nts* **(F”)** are separate. **(G)** *penka*-positive cells **(G’)** are scattered, but do not overlap with *vip* expression **(G”). (H)** *oxt*-positive **(H’)** and *cck*-positive cells **(H”)** are also separated. **(I)** The clusters formed by cells expressing *oxt* **(I’)** or *vip* **(I”)** do not show coexpression. **(J)** *vip* **(J’)** and *penkb* **(J”)** are also not coexpressed. **(K)** The rostral location of *cck*-positive cells **(K’)** is separate from the neighboring *avp*-positive cluster **(K”). (L)** *penka*-positive cells **(L’)** also are close to the *cck*-positive population **(L”)**, but still separate. **(M)** Cells positive for *penkb* **(M’)** surround the central *nts*-positive cluster **(M”). (N)** Cells expressing *avp* **(N’)** or *oxt* **(N”)** are intermingled, but the peptides are not coexpressed (single planes: **O–O”**). **(P)** Cells expressing *nts* **(P’)** or *oxt* **(P”)** have similar locations, but these peptides are not coexpressed. **(Q)** Cells expressing *oxt* **(Q’)** or *sst1.1* **(Q”)** are also intermingled and do not coexpress these peptides (single planes: **R–R”**). All images show maximum intensity projections, unless indicated otherwise. Scale bar: 50 μm.

Many of the neuropeptide combinations (16/36) showed no coexpression in the same cell, and often these neuropeptides were expressed in spatially separate clusters. The rostralmost clusters formed by cells expressing *avp*, *crh*, *cck*, or *sst1.1* did not show any overlap with the caudalmost cluster formed by cells expressing *vip* (Figures 2A–D”, 5–8 animals analyzed). Among the rostral group, *crh* and *cck* were not coexpressed in the same cells, and *cck* expression was also separate from the large *nts*-positive cluster (Figures 2E–F”, 6–7 animals analyzed). The *penka*-expressing cluster extended as far caudally as the small group of *vip*-positive cells, which however occupied a more lateral region (Figures 2G–G”, 8 animals analyzed). *oxt*-expressing cells formed a large central cluster, and they did not overlap with the rostrally located *cck*-expressing cells, nor with the caudally located *vip*-expressing group (Figures 2H–I”, 6–9 animals analyzed). The *vip*-positive cluster was also separate from cells expressing *penkb* (Figures 2J–J”, 7 animals analyzed). The rostral cluster of *cck-positive* cells was close to, but separate from the *avp*-positive and *penka*-positive clusters (Figures 2K–L”, 7 animals analyzed). *penkb*-positive cells surrounded the more central *nts*-positive cluster (Figures 2M–M”, 5 animals analyzed). Within the center of the NPO, cells expressing *avp* or *oxt* were found intermingled in the same region, but did not overlap (Figures 2N–O”, 8 animals analyzed). The central and intermingled clusters of cells expressing *oxt* or *nts* did not show coexpression of these two peptides (Figures 2P–P”, 10 animals analyzed). Similar spatial proximity without coexpression was also found for cells expressing *oxt* or *sst1.1* (Figures 2Q–R”, 8 animals analyzed).

### Variable coexpression

**Figure 3:**
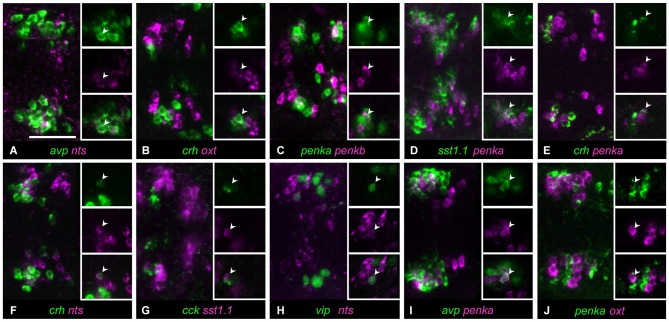
**In some cell type staining combinations, occasional and low coexpression can be observed. (A)** Cells expressing *avp* or *nts* only overlap in rare cases. **(B)** Cells expressing *oxt* or *crh* are usually separate, but occasially these peptides are coexpressed in a single cell. **(C)** Cells expressing *penkb* cells usually surround the *penka*-positive cluster, but in few animals, one cell shows coexpression. **(D)** *penka*-positive cells and *sst1.1*-positive cells are intermingled, and occasionally show low coexpression. **(E)** *crh*-positive cells are intermingled with *penka*-positive cells, but some rare occurences of coexpression were found. **(F)** *crh* can also be coexpressed with *nts* in rare cases. **(G)** *cck*-positive cells appear to faintly coexpress *sst1.1* in few of the animals. **(H)** One isolated cell was sometimes found coexpressing *vip* and *nts*. **(I)** Cells expressing *avp* or *penka* are intermingled and usually these peptides are not coexpressed, but single coexpressing cells do occur. **(J)** Stainings for *oxt* and *penka* often show no coexpressing cells, but sometimes these peptides are coexpressed in few cells. Images show maximum intensity projections, insets show split channels of single confocal planes with coexpressing cells (arrowheads). Scale bar: 50 μm.

Occasionally, a single cell was found coexpressing two neuropeptides in a larva, although most of the animals analyzed did not show coexpression. We observed such rarely coexpressing single cells in 10/36 neuropeptide combinations. A single *avp*-positive cell was occasionally found within *nts*-positive cells (Figure 3A, 3/11 animals). Similarly, in rare cases, single cells showed coexpression of *crh* and *oxt* (Figure 3B, 3/17 animals). The clusters of cells producing *penka* or *penkb* were spatially separate, but in one animal, coexpression could be found in a single cell (Figure 3C, 1/16 animals). The intermingled clusters of cells expressing *penka* or *sst1.1* also in rare cases showed one cell with coexpression (Figure 3D, 2/14 animals). The *crh*-positive cluster occupied the rostral half of the region covered by both the *penka*-positive and *nts*-positive clusters, and in rarely occuring cells, *crh* was coexpressed with *penka* (Figure 3E, 4/14 animals) or *nts* (Figure 3F, 1/6 animals). While the *cck*-positive cluster was rostral, occasional coexpression of *sst1.1* was observed (Figure 3G, 3/7 animals). The *vip*-producing cluster was caudal and lateral, but a cell that was more rostromedial did in one case coexpress *nts* (Figure 3H, 1/9 animals). The central cluster of *penka*-positive cells was intermingled with the clusters of cells expressing *avp* or *oxt*, and occasionally single *penka*-positive cells coexpressed *avp* (Figure 3I, 4/21 animals) or *oxt* (Figure 3J, 4/33 animals).

**Figure 4:**
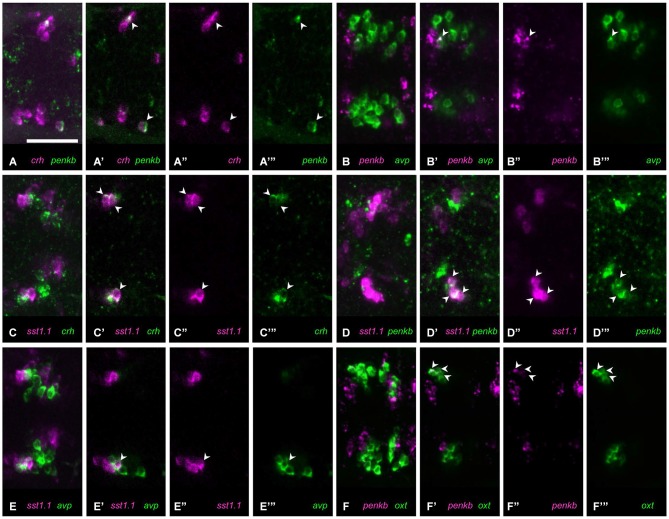
**Some peptide combinations show low or moderate coexpression. (A)** Coexpression (**(A)** maximum intensity projection; **(A’)** single plane) of *crh* **(A”)** and *penkb* **(A”’)** rarely occurs, but can be found in more than one cell. **(B)** In some animals, coexpression (**(B)** maximum intensity projection; **(B’)** single plane) of *penkb* **(B”)** and *avp* **(B”’)** can be found. **(C)** Moderate coexpression (**(C)** maximum intensity projection; **(C’)** single plane) of *sst1.1* **(C”)** and *crh* **(C”’)** can be observed in some animals. **(D)** Few cells also show coexpression (**(D)** maximum intensity projection; **(D’)** single plane) of *sst1.1* **(D”)** and *penkb* **(D”’)**. **(E)** Coexpression is found in few cells (**(E)** maximum intensity projection; **(E’)** single plane) labeled for *sst1.1* **(E”)** and *avp* **(E”’)**. **(F)** In some animals, moderate coexpression (**(F)** maximum intensity projection; **(F’)** single plane) of *penkb* **(F”)** and *oxt* **(F”’)** can be found. Arrowheads mark coexpressing cells. Scale bar: 50 μm.

**Figure 5:**
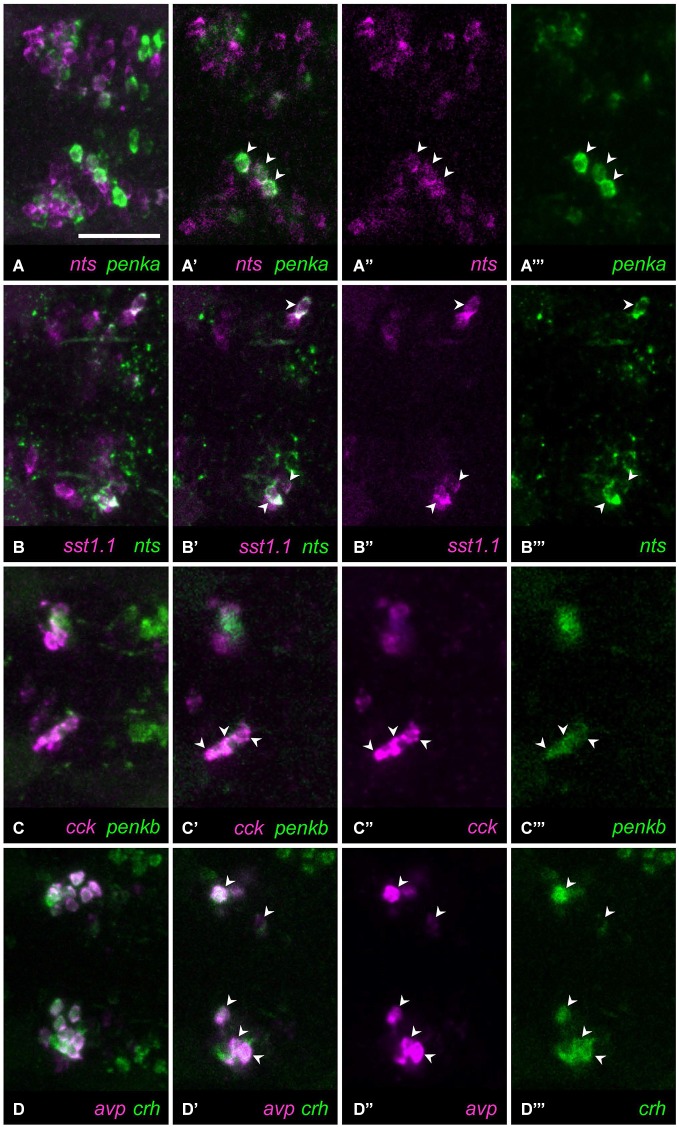
**Four combinations of neuropeptides show higher coexpression. (A)** In most animals, several cells show coexpression (**(A)** maximum intensity projection; **(A’)** single plane) of *nts* **(A”)** with *penka* **(A”’)**. **(B)** In all animals, few cells show coexpression (**(B)** maximum intensity projection; **(B’)** single plane) of *sst1.1* **(B”)** and *nts* **(B”’)**. **(C)** High coexpression can be found (**(C)** maximum intensity projection; **(C’)** single plane) in the rostral clusters of cells expressing *cck* **(C”)** or *penkb* **(C”’)**. **(D)** Consistently high coexpression (**(D)** maximum intensity projection; **(D’)** single plane) can be observed for the intermingled *avp*-positive **(D”)** and *crh*-positive clusters **(D”’)**. Arrowheads mark coexpressing cells. Scale bar: 50 μm.

The extent of coexpression for other peptide combinations was somewhat higher where more than one cell per animal showed coexpression. In the combination of *crh* and *penkb* staining, many animals showed no coexpression, but in one animal, coexpression was observed in few cells (Figures 4A–A”’, 1/12 animals). Similarly few *avp* and *penkb* coexpressing cells were found in some animals analyzed (Figures 4B–B”’, 5/15 animals). In those cases in which *crh* and *sst1.1* were coexpressed, we found such coexpression in several cells (Figures 4C–C”’, 2/9 animals). Few cells coexpressing *penkb* and *sst1.1* were also found in one animal (Figures 4D–D”’, 1/5 animals). Few *avp* and *sst1.1* coexpressing cells were also found in some animals (Figures 4E–E”’, 6/25 animals). Some animals showed few *oxt* and *penkb* coexpressing cells (Figures 4F–F”’, 4/7 animals). Although in some animals, *penka* and *nts* were not coexpressed, in most animals we found coexpression of these peptides in few cells (Figures 5A–A”’, 8/15 animals).

### Consistent coexpression in all animals

**Figure 6:**
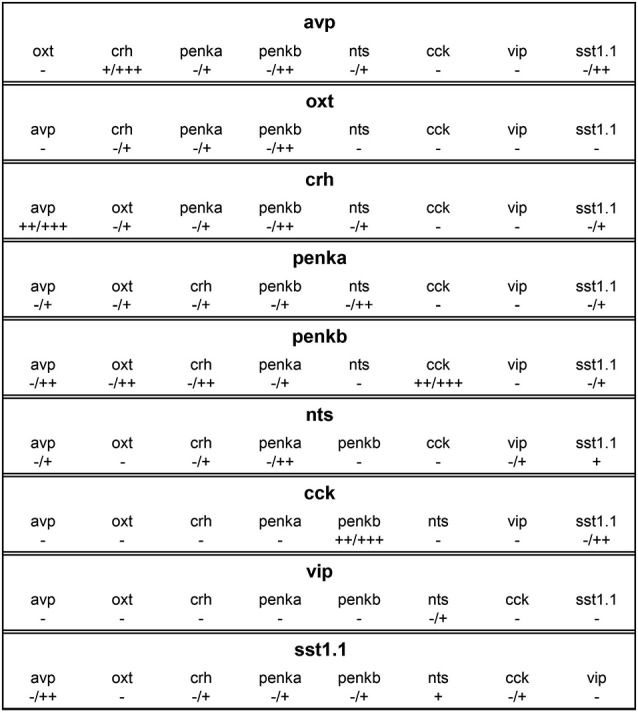
**Overview of coexpression profiles of cells expressing *avp*, *oxt*, *crh*, *penka*, *penkb*, *nts*, *cck*, *vip*, or *sst1.1***. Coexpression degrees indicate complete absence (−), rare occurrence (+), low coexpression (++), or high coexpression (+++). In many combinations, coexpression was variable, and the minimum and maximum degrees are indicated (min/max). Differences between the coexpression of one peptide in one cell type and vice versa are caused by differences in cell cluster sizes.

Consistent coexpression in all animals analyzed was found for three peptide combinations. The combination of *nts* and *sst1.1* showed consistent coexpression in few cells (Figures 5B–B”’, 5/5). High degree of coexpression was consistently found for the rostralmost clusters formed by cells producing *penkb* or *cck* (Figures 5C–C”’, 13/13 animals). Coexpression was also consistently high for the combination of *avp* and *crh* staining (Figures 5D–D”’, 19/19 animals). These results are summarized in Figure 6, in which we also indicate the range of coexpression observed (minimum and maximum degrees).

## Discussion

Our results show that many of the peptides produced by densely intermingled cells of the larval zebrafish NPO are not coexpressed. Occasional coexpression in one cell can be observed for other peptide staining combinations, and some combinations with low or moderate coexpression appear more frequently. Only three of the 36 combinations show consistent coexpression in all animals analyzed, reaching a high degree only for *avp* + *crh* and *cck* + *penkb*. It should be noted that the presence of RNA does not necessarily mean that the peptide will also be synthesized. For example, there is a distinct cluster of *avp*-expressing cells outside the NPO in the ventral hypothalamus, which can clearly be labeled by ISH, but not by immunohistochemistry (IHC; Eaton et al., 2008; Herget et al., 2014). Still, the information presented here about coexpressed peptide transcripts suggests the existence of subclasses of cell types which can have very different functions.

The large amount of information available on the degree of coexpression of different neuropeptides in the mammalian PVN allows comparisons of our results with those obtained in mammals. Interestingly, many peptide combinations show similar degrees of coexpression both in larval zebrafish and in mammals. The absence of coexpression of *avp* with *oxt* we observed is in line with rat data (Swanson and Sawchenko, 1983). The coexpression of *avp* with *crh* we found to vary between moderate and high levels was also shown to be low or high in the rat (Sawchenko et al., 1984b; Whitnall et al., 1985, 1987; Aubry et al., 1999; Arima et al., 2001; Simmons and Swanson, 2009), and was low in the mouse or sheep (Rivalland et al., 2005; Biag et al., 2012). Recently, it was found that only magnocellular CRH-positive cells coexpress AVP in the rat (Dabrowska et al., 2013). Coexpression of CRH and AVP was also found in other teleosts (Yulis and Lederis, 1987; Olivereau et al., 1988; Fryer, 1989). We found rare coexpression of *oxt* with *penka*, and low coexpression of *oxt* with *penkb*, and coexpression of OXT with ENK was also seen in the rat (Martin and Voigt, 1981; Rossier et al., 1983). OXT and SST do not overlap in the rat (Swanson and Sawchenko, 1983), and we saw the same absence of coexpression in the fish. We observed rare coexpression of *crh* with *penka*, and low coexpression of *crh* with *penkb*, while ENK coexpression with CRH seems to be higher in rats (Hökfelt et al., 1983; Ceccatelli et al., 1989; Pretel and Piekut, 1990), or sheep (Rivalland et al., 2005). However, one more recent study also found only low coexpression of ENK in CRH-producing cells in the rat (Dabrowska et al., 2013), which is more in line with our results. NTS and ENK are coexpressed in low degrees in the rat (Ceccatelli et al., 1989), and we also saw consistent coexpression of *nts* with *penka*, but not with *penkb*. It was reported that very few NTS cells coexpress VIP in the rat (Ceccatelli et al., 1989), and the coexpression of *nts* and *vip* was also rare here.

Other peptide coexpression profiles we observed in larval zebrafish deviate from previously established mammalian data. AVP and ENK are thought to be coexpressed in the rat (Martin and Voigt, 1981), and moderately coexpressed in the sheep (Rivalland et al., 2005), but *penka* was only rarely coexpressed with *avp*, and *penkb* showed only low coexpression with *avp* in our case. We did not find coexpression of *avp* with *cck*, but there are many such cells in the rat (Mezey et al., 1986). Our observation showed low coexpression of *avp* with *sst1.1*, but those are non-overlapping cell types in the rat (Swanson and Sawchenko, 1983). *oxt* coexpression with *crh* was rare here, but variably low or high in the rat (Sawchenko et al., 1984a; Dabrowska et al., 2013), and low in the mouse (Biag et al., 2012). In the zebrafish, one study reported colocalization of *oxt* and *crh* in the preoptic area (Chandrasekar et al., 2007), but with our increased resolution, we can clarify that those cells only reside in the same region, and are in fact intermingled. OXT and CCK are coexpressed in the rat (Vanderhaeghen et al., 1981; Bondy et al., 1989; Levin and Sawchenko, 1993), but we saw no such coexpression in the zebrafish. CRH coexpression with NTS was reported to be low (Sawchenko et al., 1984a) or moderate in the rat (Ceccatelli et al., 1989), but according to our results coexpression only occurs rarely. CRH and CCK are coexpressed in some cells in the rat (Mezey et al., 1986; Ceccatelli et al., 1989), but not at all in our data. CRH and VIP were reported to be coexpressed in low levels in the rat (Ceccatelli et al., 1989), but they are not coexpressed here. ENK and VIP are coexpressed in the rat (Hökfelt et al., 1987), but neither *penka* nor *penkb* overlap with *vip* in our results. NTS and CCK show very low coexpression in the rat (Ceccatelli et al., 1989), but no coexpression in our results. All observed differences can originate from differences in both species and age, since we compare larval zebrafish data with adult mammalian results. Changes in coexpression levels during continued development from the larval stage detailed here into adulthood can also be expected.

To our knowledge, several peptide combinations were never addressed as far as coexpression is concerned. *avp* coexpression with *nts* was not discussed before, and rarely occurs here. Also, *crh* coexpression with *sst1.1* was not addressed before, and is rare here. We found that *penka* is not coexpressed with *cck*, which was not addressed before. Coexpression of *penkb* with *cck* was high, and is reported here for the first time. Similarly, coexpression of *penka* or *penkb* with *sst1.1* was not reported before, and we found rare or low coexpression. Also the coexpression of *sst1.1* and *nts* is shown here for the first time. Coexpression of *cck* and *vip* was never addressed before, and we found that they do not coexpress. We also report rare coexpression of *cck* with *sst1.1* for the first time. Lastly, we also show that the expression of *vip* does not overlap with that of *oxt* or *sst1.1*.

The rarely observed coexpression in only few cells or few animals is a deviation from the otherwise complete absence of coexpression seen in most cells or animals for these combinations of stainings. Potential causes of such rare coexpression could be transitional stages in the development of these cells, mismapping due to lack of resolution, or stochastic variation. The confocal microscopy used allowed sufficient depth resolution to spatially separate cells in all three dimensions and therefore mismapping is unlikely.

The high degree of coexpression of *avp* and *crh* observed has immediate functional implications. Similar to CRH, AVP stimulates adrenocorticotropic hormone (ACTH) secretion (Gillies and Lowry, 1979; Rivier and Vale, 1983). A wide range of coexpression levels was reported in different fish species. Coexpression of *avp* and *crh* was very high, reported as 100%, in *Catostomus*, but absent in *Anguilla* (Yulis and Lederis, 1987; Olivereau and Olivereau, 1990). In humans, AVP/CRH coexpression increases with age, and a connection with stress has been implied (Raadsheer et al., 1993). In addition to CRH and AVP, CCK can also stimulate ACTH release, and CCK acting in concert with AVP has a similar ACTH-releasing potency as CRH alone (Mezey et al., 1986). We saw no coexpression of *cck* with *avp* or *crh*. In contrast to CRH, AVP, and CCK, OXT generally inhibits stress responses, while local, endogenous OXT potentiates hypothalamo-pituitary-adrenal (HPA) axis activity, suggesting a dual mechanism of OXT released within the PVN (Neumann, 2002). In our results, coexpression of *oxt* and *crh* was very rare, and only present in single cells when it occurred. The spatial proximity of the densely intermingled cells positive for *oxt* or *crh* suggests that local release of Oxt could have immediate effects on *crh*-producing cells. ENK, CRH, and AVP were found to be colocalized within the same secretory vesicles (Hisano et al., 1987), suggesting that coexpressed peptides are packaged into common vesicles and coreleased at the synapse. We found that either *penka* or *penkb* can be coexpressed in both *crh*-positive and *avp*-positive cells. The concerted action of coreleased CRH and ENK is thought to fine-tune stress regulation (Pretel and Piekut, 1990).

In the rat, CRH-positive innervation of the neurohypophysis was suggested by observations of CRH in neurohypophyseal terminals (Bloom et al., 1982). The coexpression of CRH in magnocellular cells innervating the neurohypophysis could allow direct CRH release to the general circulation. The stress axis is also affected by VIP (Westendorf et al., 1983; Tilders et al., 1984), and NTS (Gudelsky et al., 1989). In the parvocellular PVN, coexpression of NTS and CRH was found, and the fractions of CRH-positive cells that coexpress AVP or NTS form different discrete subsets (Sawchenko et al., 1984a). The reported segregation of cells expressing AVP or CRH in rats cannot be found in larval zebrafish, and *vip* is not coexpressed with *crh* here, but we did find coexpression of *nts* and *crh*.

The CRH-producing cells coexpressing AVP were reported to be stress-responsive, while the AVP-negative CRH-producing cells do not respond to stress (Whitnall, 1993). Under osmotic stress, ENK and CRH levels are increased, but restraint and swimming stress only elevated CRH, not ENK (Harbuz and Lightman, 1989). Such findings demonstrate the functional implications of coexpression differences. For a cell, switching on the coexpression of another peptide is an elegant strategy to change its function without the need for major structural rearrangement of input or target projections, which could impose much stronger metabolic demands. Neurochemical switching in response to alterations in environmental conditions has been suggested as a relevant biological phenomenon in PVN neurons (Kiss, 1988; Swanson, 1991). Dynamic adaptation of neuroendocrine transcription to altered supply or demand for neuropeptides has also been suggested in zebrafish larvae (Kurrasch et al., 2009). With the coexpression profiles established here for the larval zebrafish under basal conditions, alterations triggered by environmental challenges can be studied in a model organism that has important advantages for the analysis of neural structure and function in intact and genetically tractable animals.

## Conflict of interest statement

The Guest Associate Editor Gonzalo Alvarez-Bolado and Review Editor Matthias Carl declare that, despite being affiliated to the same institution as author Ulrich Herget, the review process was handled objectively and no conflict of interest exists. The authors declare that the research was conducted in the absence of any commercial or financial relationships that could be construed as a potential conflict of interest.
